# An integrated view of the role of miR-130b/301b miRNA cluster in prostate cancer

**DOI:** 10.1186/s40164-018-0102-0

**Published:** 2018-05-02

**Authors:** Rafael Sebastián Fort, Cecilia Mathó, Carolina Oliveira-Rizzo, Beatriz Garat, José Roberto Sotelo-Silveira, María Ana Duhagon

**Affiliations:** 1 Laboratorio de Interacciones Moleculares, Facultad de Ciencias, Universidad de la República, Montevideo, Uruguay; 2 Depto. de Genética, Facultad de Medicina, Universidad de la República, Montevideo, Uruguay; 3 Depto. de Genómica, Instituto de Investigaciones Biológicas Clemente Estable, Ministerio de Educación y Cultura, Montevideo, Uruguay; 4 Depto. de Biología Celular y Molecular, Facultad de Ciencias, Universidad de la República, Montevideo, Uruguay

**Keywords:** hsa-miR-301b, hsa-miR-130b, TCGA, miRNA, Prostate, Cancer, DNA Methylation, Transcriptome, Microarray, Clinical outcome

## Abstract

Prostate cancer is a major health problem worldwide due to its high incidence morbidity and mortality. There is currently a need of improved biomarkers, capable to distinguish mild versus aggressive forms of the disease, and thus guide therapeutic decisions. Although miRNAs deregulated in cancer represent exciting candidates as biomarkers, its scientific literature is frequently fragmented in dispersed studies. This problem is aggravated for miRNAs belonging to miRNA gene clusters with shared target genes. The miRNA cluster composed by hsa-mir-130b and hsa-mir-301b precursors was recently involved in prostate cancer pathogenesis, yet different studies assigned it opposite effects on the disease. We sought to elucidate the role of the human miR-130b/301b miRNA cluster in prostate cancer through a comprehensive data analysis of most published clinical cohorts. We interrogated methylomes, transcriptomes and patient clinical data, unifying previous reports and adding original analysis using the largest available cohort (TCGA-PRAD). We found that hsa-miR-130b-3p and hsa-miR-301b-3p are upregulated in neoplastic vs normal prostate tissue, as well as in metastatic vs primary sites. However, this increase in expression is not due to a decrease of the global DNA methylation of the genes in prostate tissues, as the promoter of the gene remains lowly methylated in normal and neoplastic tissue. A comparison of the levels of human miR-130b/301b and all the clinical variables reported for the major available cohorts, yielded positive correlations with malignance, specifically significant for T-stage, residual tumor status and primary therapy outcome. The assessment of the correlations between the hsa-miR-130b-3p and hsa-miR-301b-3p and candidate target genes in clinical samples, supports their repression of tumor suppressor genes in prostate cancer. Altogether, these results favor an oncogenic role of miR-130b/301b cluster in prostate cancer.

**Electronic supplementary material:**

The online version of this article (10.1186/s40164-018-0102-0) contains supplementary material, which is available to authorized users.

## Background

Worldwide, prostate cancer (PrCa) is the second most frequently diagnosed cancer and the sixth major cause of cancer-related deaths in men. Although the disease frequently evolves slowly, remaining indolent for years, a minority of patients will rapidly progress to a very aggressive form which is resistant to castration therapy. The lack of reliable biomarkers, able to identify the high-risk patients who will really benefit from an intensive treatment, leads to unnecessary interventions that increase patient morbidity and health costs. Limiting the medical intervention to an active surveillance of low-risk patients would be the desirable once improved PrCa biomarkers become available.

MicroRNAs (miRNAs) are endogenous small non-coding RNA molecules (19–22 nucleotides in length) capable to modulate protein levels through their sequence-specific interaction with target mRNAs [1, 2]. Diverse studies have revealed that miRNA regulation has a wide impact in human gene expression and variation [2, 3], thus miRNAs are known to be extensively deregulated in cancer [4, 5] including prostate [6–8]. The miRNA molecular nature, tissue specificity, stability and availability in several body fluids, together with the advent of sensible and reliable quantification methods, make them outstanding candidates as biomarkers in PrCa [9, 10].They are also potentially valuable therapeutic targets [11]. Although there is a large amount of literature about specific miRNAs deregulated in PrCa, a unified picture of their function and their clinical value is still incomplete; this is more challenging for miRNAs families and clusters which are expected to co-target common sets of mRNAs [12]. An improved understanding of their definite role as driver or passenger genes and their molecular targets in PrCa is needed. More importantly, appropriate validations using independent cohorts, as well as larger prospective studies are needed to achieve a more precise picture of their clinical utility [13].

Mounting evidence across several cancer types, including breast [14], bladder [15], glioblastoma [16], lung [17], ovarian [18], pancreatic [19] supports the involvement of the miR-130b/301b gene cluster in carcinogenesis. Indeed, this family belongs to a superfamily that has been proposed as a pan-cancer oncogenic miRNA superfamily that targets prominent tumor suppressor genes (TSGs), such as TGFBR2, SMAD4, PTEN [20]. However, the role of its derived miRNAs in cancer seems to be tissue specific, as it has been shown to display both oncogenic or tumor suppressor functions.

Here we sought to progress into the understanding of the clinical significance of the miR-130b/301b cluster in PrCa through the unified analysis of most of the published literature and the available PrCa gene expression datasets (small RNA and mRNA transcriptomic, methylomic and clinical data). First, we compiled the published studies showing a deregulation of both miRNAs in independent PrCa cohorts and the clinical value assigned in the original articles. Then, we performed new analyses of publicly available PrCa datasets, with emphasis in the largest cohort such as The Cancer Genome Atlas (TCGA-PRAD) to collect further evidence of the role of miR-130b/301b cluster in this disease. We also discuss the existing functional studies about these miRNAs with emphasis in the validated target genes that have been reported in the cancer literature. Overall, our findings stand for the upregulation of both miRNAs in PrCa, which ultimately favors an oncogenic action of the cluster in this disease.

## Methods

### Analysis of PrCa miRNA datasets

For all the PrCa studies analyzed, relevant features used in the analyses are listed in Table 1. Depending on the type of study and the availability of the data, we followed different strategies. Data deposited at GEO was analyzed using the GEO2R tool using default settings [21], selecting the samples by clinical status definition. Data not available in repositories was extracted from the original article. Martens-Uzunova et al. RNA-seq data [22] was provided by the authors; it was normalized, counted and annotated using miRDeep2 software using default parameters [23] and miRBase database Release 21. The small RNA-seq data from the TCGA-PRAD was retrieved as explained in the next section.

**Table 1:** miR-130b/301b expression in PrCa datasets

Repository	Disease condition	Sample type	miR	Deregulation	Fold change	*p v*alue	PMID
GSE65061	Early/late recurrence	FFPE tissue	301b	UP^4^	1.7	0.026	25760964
GSE26245	Recurrent/non-recurrent	FFPE tissue	130b	UP^4^	1.2	0.024	21703393
GSE26247	Recurrent/non-recurrent	FFPE tissue	130b	UP^4^	1.3	0.017	21703393
Personal communication	Metastatic/primary tumor	Frozen tissue	130b	UP^4^	1.6	NA	21765474
GSE35813	PC-3/RWPE-1	Cell lines	130b and 301b	UP^4^	1.7 and 1.5	0.011 and 0.0091	22982408
GSE80400	Tumor/normal	Frozen tissue	130b	UP^4^	1.9	0.14	26041889
GSE21032, GSE21036, E-TABM-794	Tumor/normal Recurrent/non-recurrent	Frozen tissue	130b	UP^3^	NA NA	NA 0.004	26489476
GSE34933, GSE21032, GSE26367	Tumor/normal	Frozen and FFPE tissue	130b	UP^3^	NA	0.00000035	27179774
GSE21036 (PMID 20579941)	Primary tumor/normal Metastatic/primary tumor Metastatic tumor/normal	Frozen tissue	130b	UP^3^	1.5 1.7 2.6	< 0.0001 = 0.0002 0.0001	28192399
GSE55323, GSE26245, GSE26247, GSE65061, GSE62610, GSE46738	Recurrent/non-recurrent	Frozen and FFPE tissue	301b	UP^3^	NA	0.0066	28651018
GSE36803	Tumor/normal	Frozen tissue	130b	UP^2^	< 1.5	0.0019	23233736
NA	Tumor/normal	FFPE tissue	130b	UP^2^	2.2	0.02	27120795
NA	Resistant/sensitive to docetaxel	Cell lines	301b	UP^1^	2.2	0.002	24714754
GSE14857	Tumor/normal	Frozen tissue	130b	UP^1^	1.5	0.0006	19676045
E-TABM-794	Tumor/normal	Frozen tissue	130b and 301b	UP^1^	> 1.5	< 0.05	21765474
NA	PrCa patients/Normal control individuals PrCa metastatic/PrCa localized patients	Plasma	130b	UP^1^	4.7 6.1	0.034 0.007	22240788
E-MTAB-408	Primary tumor/BPH	Frozen tissue	130b	UP^1^	2.00	0.00019	22266859
E-MTAB-408	CRPCs/BPH	Frozen tissue	130b	UP^1^	2.5	0.011	22266859
NA	C4-2B/RWPE-1 C4-2B/RWPE1 derived exosomes	Cell lines	130b	UP^1^	~ 2, 3.7	< 0.05	24715691
GSE62610	Recurrent/non-recurrent	FFPE tissue	301b	UP^1^	2	0.025	25416653
NA	Exosomes/cell line	Cell lines	130b	UP^1^	8.6	NA	27102850
NA	Short/long overall survival	FFPE tissue	301b	UP^1^	NA	0.029	27307749
NA	Hypoxia/normoxia	Cell lline	301b	UP^1^	> 2	NA	27327120
NA	Patients/healthy donors	Exosomes	130b	UP^1^	NA	0.0021	28192399
NA	Recurrent/non-recurrent	Tissue	130b	UP^1^	NA	0.018	28192399
NA	After/before docetaxel	Plasma and serum	301b	UP^1^	1.6	0.04	24714754
GSE46738	High/Low pre-operatory PSA levels	FFPE tissue	130b and 301b	DOWN and UP^1^	− 1.3 and 1.2	< 0.049 and < 0.003	25663948
GSE55323	Recurrent/non-recurrent	Tissue	301b	DOWN^4^	− 1.2	0.017	24967583
NA	Tumor/normal	FFPE tissue	130b	DOWN^1^	NA	< 0.0001	25154741
NA	Gleason 4 or 5/gleason 3 Recurrent/non-recurrent	Serum	130b	DOWN^1^	NA	< 0.05	25874774
GSE52955	Tumor/normal	Frozen tissue	130b and 301b	DOWN^1^	NA	< 0.0001 and 0.0014	28166834

Disease condition and sample type are named according to the original study. References to the publications are presented as PMID. Data repository IDs: GSE corresponds to data deposited in Gene Expression Omnibus (GEO), and E-XXXX-XXX corresponds to data stored in Array Express. The fold change in expression between the conditions with its correlative *p* value were obtained from diverse sources which are indicated with superscript numbers in column “Change” as follows: 1. Identified and selected by the original publication, 2. Identified but not selected by the original publication, 3. Identified by analysis of the original study by others, 4. Identified in the present study by analysis of the original report

### Analysis of prostate transcriptomic and clinical profiles of TCGA-PRAD

Data on mRNA expression, miRNA expression as well as clinical information (when available) from PrCa and matched normal patient samples generated by The Cancer Genome Atlas (TCGA) consortium were retrieved from UCSC Xena Browser [24]. It comprises mRNAseq Level_3 data (file names: *.rsem.genes.normalized_results) of 550 samples, miRNAseq data Level_3 data (file names: *.isoform.quantification.txt) of 544 samples.

### Analysis of PrCa DNA methylation data

The DNA methylation data of the TCGA-PRAD cohort, obtained using Illumina Infinium Human Methylation 450 BeadChip arrays of the 50-paired normal and prostate tumor samples and additionally unmatched normal and tumor tissues (498 in total) was extracted using FIREBROWSE [25]. Several public methylomes available at the Gene Expression Omnibus (GEO) database [26, 27] were also analyzed: PrCa clinical datasets GSE38240 [28], GSE52955 [29] and GSE76938 [30] and PrCa cell lines GSE34340, GSE62053, GSE54758 [31, 32]. The normalized average beta-values for the following miR-130b/301b 12 CpGs were calculated: cg13879495, cg04378107, cg22678932, cg12155013, cg14030055, cg11673244, cg02473781, cg16244770, cg04282607, cg16974014, cg03636163, cg03328201.

### Statistical analysis

The corresponding variables are expressed as average value ± standard deviation (SD). Statistical analyses were done using two-tailed t test, and the statistical significance of the observed differences were expressed using the *p* value (*p*). D’Agostino-Pearson was conducted as the normality test and nonparametric Spearman was used to test correlations.

## Results

### The genomic and epigenomic context of the miR-130b/301b cluster support their coordinated expression in PrCa

**Fig. 1:**
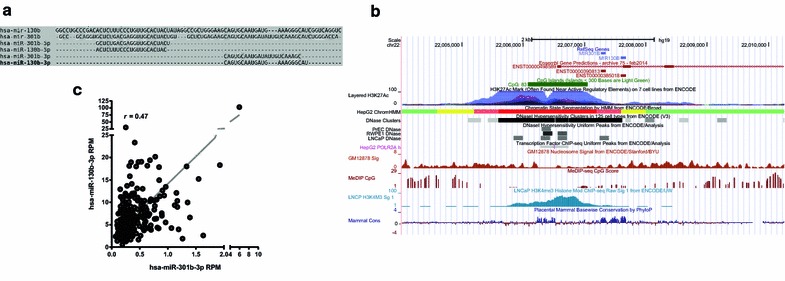
Genomic and epigenomic context of the human miR-130b/301b gene cluster. **a** Sequence alignment of the miR-130b/301b gene cluster. miRNAs precursor (hsa-mirs) and mature miRNAs (hsa-miRs) are indicated. Sequences were retrieved from miRBase and the alignment was performed in SeaView using CLUSTALW with default parameters. **b** Genomic view of the miR-130b/301b gene cluster region in UCSC Genome browser (GRCh37/hg19) centered at the transcription star point of the di-cistronic transcript. Several ENCODE tracks are displayed (see the text for explanation). **c** Co-expression of miR-130b and miR-301b in prostate clinical samples of TCGA-PRAD, including normal and tumor tissue. Correlation between the levels of miR-301b (x axis) and miR-130b (y axis) of the TCGA-PRAD cohort. n = 294 (samples that report expression data for miR-301b). Data was obtained from RNA-seq of small RNAs downloaded from UCSC Xena Browser and is expressed in reads per million (RPM). The non-parametric Spearman correlation coefficient (*r*) is indicated

miR-130 gene family (miRbase record MIPF0000034) [33] is vertebrate specific. In the human genome, it is composed by four miRNA precursor genes: mir-301a (at chr17), mir-130a (at chr11), mir-130b and mir-301b (at chr22). The miRNAs derived from the miR-130b/301b gene cluster precursors share an identical seed region (Fig. 1a). The precursor RNAs hsa-mir-130b and hsa-mir-301b are processed preferentially from the 3′ arm of the hairpin to generate mature miRNAs hsa-miR-130b-3p and hsa-miR-301b-3p (hereafter referred as miR-130b and miR-301b, respectively). Their current transcript annotation suggests that they are transcribed as a di-cistronic RNA transcript composed of 7 noncoding exons, which is classified as a “known processed transcript” [34]. The transcript has been manually annotated by HAVANA project, and its status (“known”) indicates that it is identical to known cDNAs. However, it is assigned a support level 7, meaning that no single transcript supports the model structure, therefore its current structure is still speculative. The stem-loop precursors of miR-130b and miR-301b are coded 327 bp apart, where mir-301b is in the first intron of the transcript (chr22:22007270-22007347 of GRCh37/hg19 or chr22:21652981-21653058 of GRCh38/hg38) and mir-130b spans from the first intron to the beginning of exon 2 (chr22:22007593-22007674 of GRCh37/hg19 or chr22:21653304-21653385 of GRCh38/hg38) (see Fig. 1b and Additional file 1: Figure S1 for a detailed view of the region). Regulatory features of the transcript, identified by ENCODE Project [35], including DNA accessibility (DNaseI hypersensitivity clusters), DNA methylation (CpG islands), chromatin status (H3K27Ac and H3K4M3 marks, nucleosome positioning by MNaseI), polymerase and transcription site binding (ChIP-seq), and histone modification, suggest that the cluster is controlled by a unique upstream promoter (Fig. 1b and Additional file 1: Figure S1 for a detailed view) whose transcription start point (TSS) is located at chr22:22006559 of GRCh37/hg19 (chr22:21652270 of GRCh38/hg38). Specifically, the PrCa cell lines’ data compiled in the browser shows that DNaseI hypersensitivity patterns (LNCaP, PrEC, RWPE-1) as well as H3K4me3 histone deposition sites (LNCaP) is consistent with RWPE-1, supporting the activity of this promoter in prostate tissue. Finally, the conservation of the region surrounding the TSS and the regions encoding both precursor RNAs supports the functionality of these sequences in vertebrates (see 100 Vert. conservation track on Fig. 1b).

The genomic and epigenomic structure of the miR-130b/301b cluster indicate that both miRNAs are co-regulated at the level of transcription. Backing this hypothesis, the levels of both miRNAs show a positive correlation in the tissue samples analyzed in the TCGA-PRAD cohort (*r* 0.47, *p *< 0.0001) (Fig. 1c). Of note, miR-301b is about ten time less abundant than miR-130b in prostatic tissue (Fig. 1c), and this probably explains why the former is less reported in the literature. As an example, only 54% (294) of the TCGA-PRAD samples detect expression value of miR-301b (Fig. 1c).

### The expression of miR-130b and miR-301b increases in PrCa neoplastic tissue and metastasis

To date, many miRNA expression profiling studies in PrCa have been published. They differ in sample size, sample nature (cell lines, prostatectomy, biopsy, exosome fraction, circulating RNA), sample stage (primary vs metastatic, clinical parameters such as Gleason Score, PSA level, biochemical recurrence), control sample (surrounding normal tissue, other normal tissue, benign prostatic hyperplasia (BPH)), quantification assay (RT-qPCR, microarray, RNA-seq) and statistical method applied. Most of them performed a genome wide quantification followed by a selection of a group of miRNAs for further analysis. We thoroughly revised the PrCa literature and found 25 independent articles in which a differential expression of miR-130b or miR-301b is recognized. From these datasets, we withdrew 31 comparisons, including those present in the original studies and those derived from subsequent analysis, and the relevant findings are summarized in Table 1. Most of the comparisons (27 out of 31) reveal an upregulation of the expression of one or both miRNAs in PrCa. The level of increase in miRNA abundance in malignant samples is 1.2–8.6 folds for miR-130b (*p* 0.05 to < 0.0001) and 1.5–2.2 folds for miR-301b (*p* 0.025 to < 0.0066). They are upregulated in primary tumor vs normal [22, 36–44], metastatic vs primary tumor [22, 38, 42], as well as recurrent vs non-recurrent patients [38, 45–48]. In addition, both miRNAs are also found upregulated in docetaxel resistant PrCa cells [49], malignant cell line PC-3 derived exosomes [50], and hypoxic PrCa cell lines [51]. On the contrary, 5 studies found that either miR-301b, miR-130b or both are downregulated in PrCa, including malignant tissue [29, 52], high Gleason Score tumors [53], high pre-operatory PSA levels [54] and recurrent patients [53, 55].The magnitude of downregulation in these cases is − 1.2 to 1.3 fold (*p* between 0.05 and < 0.0001).

**Fig. 2:**
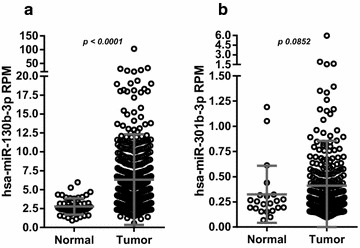
Expression of miR-130b and miR-301b in normal and tumor prostate tissue. **a** Expression of miR-130b in prostate tissues obtained from small RNA-seq data of TCGA-PRAD cohort (normal = 52, tumor = 492). **b** Expression of miR-301b in normal and tumor (normal = 22, Tumor = 273) prostate tissues obtained from small RNA-seq data of TCGA-PRAD cohort

Since the largest available dataset, the TCGA-PRAD cohort has not been interrogated for the miR-130b/301b cluster yet, we examined the levels of both miRNAs in normal vs tumor prostate tissue samples. We found a significant increase in miR-130b abundance in tumor tissue, with a median change of 2.02-fold (*p* < 0.0001) (Fig. 2a). Meanwhile, miR-301b shows a tendency to be upregulated in unmatched tumor vs normal tissue (1.20-fold, *p* 0.0852); its low abundance may explain the lack of significance of the change (Fig. 2b).

### The miR-130b and miR-301b are expressed in PrCa and their abundance is positively associated with the DNA methylation of their locus

The molecular basis for the increment of miR-130b and miR-301b expression in tumors is currently unknown. We sought to examine the available structural and regulatory features of the genes to reveal putative causes of their deregulation in cancer. The examination of nucleotide and structural variation of the region in the TCGA-PRAD (492 samples) using cBioPortal [56] only identifies 5 copy number variants for miR-301b (1 amplification and 4 deep deletions), suggesting that DNA sequence changes are unlikely to be responsible for the upregulation of this cluster observed in tumors. We then analyzed the epigenetic information obtained by independent genome-wide projects [35] that is compiled in the major human genome browsers (Fig. 1b and Additional file 1: Figure S1). The results evidence that the DNA region around the TSS is accessible, exhibiting an active chromatin state depleted of nucleosomes. In agreement with an active promoter status, a 1200 bp long CpG island marked by the deposition of RNA polymerase II and key transcription factors is present at the promoter region. Altogether, these data indicate that the promoter of the cluster is in an open chromatin state, permissive for transcription initiation. That also holds true for the three PrCa cell lines included in the ENCODE study, which show DNaseI hypersensitive peaks around the TSS and H3K4me3 modifications associated with active transcription of the nearby genes (Additional file 1: Figure S1).

**Fig. 3:**
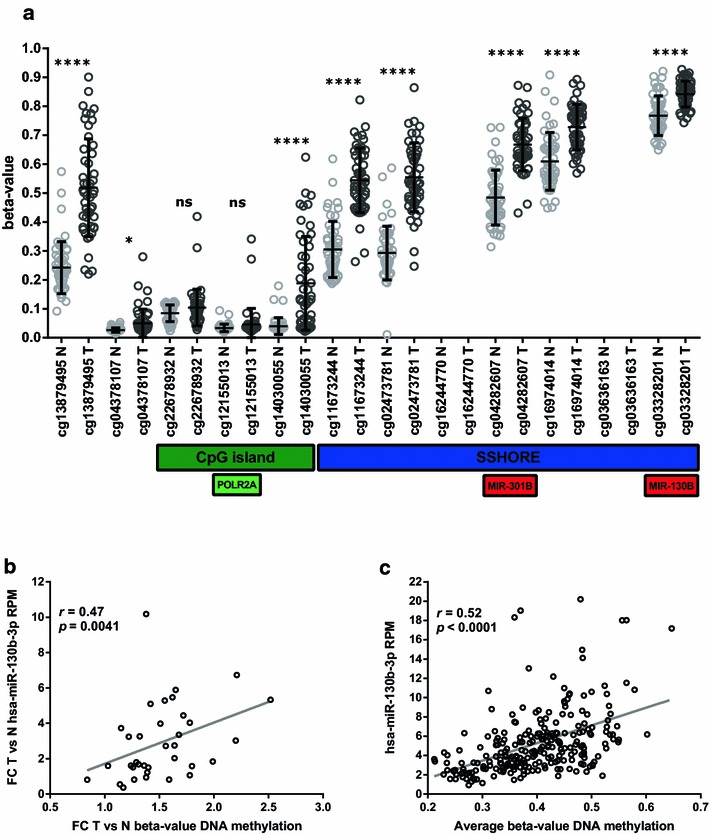
Pattern of DNA methylation of the miR-130b/miR-301b locus in prostate tissue of TCGA-PRAD. **a** Methylation levels (beta-value) of the 12 CpG dinucleotide probes located along the gene obtained using the Infinium HumanMethylation450 BeadCHiP array. The beta-value of methylation of each site from 50 normal and 50 matched tumor samples is plotted as grey (normal) and black (tumor) circles. Mean and standard deviation of the measurements are presented. The ratio of fluorescence intensity between the unmethylated and methylated sites ranges between 0 and 1 respectively. Horizontal boxes indicate the position of the CpG island, S-shore, precursor miRNAs and POLR2A (RNA Polymerase II). Data for cg16244770 and cg03636163 is not available at TCGA-PRAD. **b** Relationships between fold change (FC) in DNA methylation and miR-130b expression in matched normal vs tumor tissue (n = 35), *r* 0.4736, *p* 0.0041 **c** Relationships between DNA methylation and miR-130b expression in all available samples (n = 241), *r* 0.5242 and *p* < 0.0001. The non-parametric Spearman correlation coefficient (*r*) is indicated. *p < 0.05; **p < 0.01; ***p < 0.001; ****p < 0.0001; *ns* non-significant

Secondly, we interrogated publicly available DNA methylation array data of PrCa samples. We analyzed the available TCGA-PRAD methylation arrays of matched normal and tumor tissue (*n *= 50). The methylation pattern is similar in both tissues: an unmethylated promoter region (average beta-value < 0.2), comprising the CpG island, is followed by an intermediately methylated S-shore containing the two miRNA genes (Fig. 3a). In support of an active transcription of the locus, the CpG site located at the transcription start site(TSS)/POLR2A binding site (cg12155013) is poorly methylated (average β-value < 0.05), not showing differences between normal and tumor samples.

An increase in DNA methylation (also referred as DMM for differential methylation *m*eans) in the neoplastic tissue compared to its normal counterpart is observed throughout the locus, as evidenced by 8 significant differentially methylated CpG sites (7 *p* < 0.0001 and 1 *p* < 0.05) out of the 10 sites analyzed. Additionally, 2 out of 3 methylated sites located at the CpG island remain unchanged. It is worth to note that several CpG sites present an increased methylation variability (represented by the SD of the distribution of methylation) in tumor vs normal tissue. An increased variability in DNA methylation (also referred to as DMV for *d*ifferential *m*ethylation *v*ariation) has been reported across cancer types (Hansen et al. [57], and later validations by independent groups using TCGA and other cohorts). Moreover, it is known that the majority of DMM sites identified by comparing normal vs tumor tissue are also DMV sites [58]. In agreement with these findings, several DMMs of the miR-130b/301b locus show DMVs with higher variation in tumor compared to normal tissue (Fig. 3a and Additional file 2: Figure S2).

The same analysis was performed in three other independent PrCa cohorts (GEO accession GSE52955 [29], GSE38240 [28], GSE76938 [30] in Additional file 2: Figure S2) yielding results almost identical to those obtained with TCGA-PRAD. We then analyzed PrCa cell line methylation datasets deposited at GEO (Accession: GSE34340 [31], GSE62053 [32] and GSE54758). In agreement with TCGA-PRAD, the miR-130b/301b cluster is globally less methylated in non-malignant PrECs and RWPE-1 than in malignant cell lines (LNCaP, DU145 and PC-3) and the methylation of the CpG island at the promoter is invariably low (Additional file 3: Figure S3).

Although the epigenomic features of the region predict its active expression in normal and neoplastic prostate tissue, the global increase of its average DNA methylation in cancer may affect transcription. To assess this possibility, we used the TCGA-PRAD samples to determine if DNA methylation is associated with miR-130b level in the tissue. We observed a positive correlation for matched normal vs tumor tissue samples (*r* 0.4736, n = 35 shown in Fig. 3b) and for all the samples in the dataset (*r* 0.5242, n = 241, shown in Fig. 3c). These results favor a positive effect of DNA methylation on the transcription of this locus.

### The expression of miR-130b and miR-301b associates with negative PrCa patient clinical outcome

As described above, the deregulation of miR-130b and miR-301b has been observed in several PrCa miRNA profiling studies, with the majority of them showing an upregulation of the cluster expression in malignancy as depicted in Table 1. Several studies evidenced a positive association of miR-130b/301b cluster expression and clinicopathological parameters, comprising cancer disease diagnosis [22, 38, 40–42], presence of local and distant metastasis [59], disease stage [59], Gleason Score [59], pre-operatory PSA [54], disease recurrence [44–48, 55] and patient overall survival [38, 59]. Both miRNAs were also included in miRNA predictors that distinguish normal from cancer samples and forecast the postoperative patient outcome [22]. Yet, a small proportion of studies have reported a negative association between miR-130b/301b expression and tumor status [29, 52] (miR-130b and miR-301b), preoperatory PSA [54] (miR-130b) and biochemical recurrence (miR-130b in serum in [53] and miR-301b in tissue [55]).

**Fig. 4:**
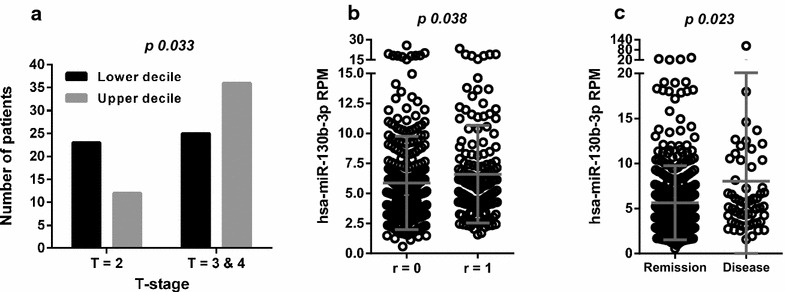
Association between the expression of miR-130b and clinical characteristics in the TCGA-PRAD cohort. **a** Comparison of pathological T-stage in patients with levels of miR-130b in the upper and lower deciles of expression (n = 96, Fisher’s exact test). **b** Level of miR-130b in patients without (R = 0 median = 5.00) or with residual tumor (R = 1 median = 5.65) (n = 456, Mann–Whitney test). **c** Level of miR-130b in PrCa tissue of patients in which the primary therapy outcome is complete remission indicated as Remission (median = 4.45) or Disease-including stable and progressive disease—(median = 5,36) (*n *= 438, Mann–Whitney test)

To further investigate the role of the miR-130b/301b cluster in PrCa we assessed its clinical value interrogating TCGA-PRAD. We analyzed the putative association between the expression of miR-130b and miR-301b and the clinical data, including preoperative PSA, Gleason Score, number of positive lymph nodes, pathological N-stage, pathological T-stage, residual tumor, primary therapy outcome success and biochemical recurrence. For every variable we assessed the total and the segmented cohort (deciles and quartiles). We did not find any negative association between the upregulation of the miRNAs and the clinical variables scored in the TCGA-PRAD cohort. On the contrary, we observed a positive association between the level of miR-130b, pathological tumor stage (Fig. 4a), residual tumor [60] (Fig. 4b) and primary therapy outcome success (Fig. 4c). Meanwhile, we were unable to find any significant association between miR-301b tissue abundance in PrCa and the clinical presentation of the disease.

### The correlation between the expression of validated direct targets of miR-130b and miR-301b supports their oncogenic function in PrCa

As discussed above, a list of empirically validated miR-130b/301b direct targets, comprising both TSG and oncogenes (OG) has been proposed. These candidates are expected to correlate negatively with the expression of miR-130b or miR-301b in PrCa if the oncogenic hypothesis holds true in the clinical samples. Given the availability of large genome wide mRNA and miRNA expression data in the TCGA-PRAD, we determined their correlation to evaluate the proposed direct targets. We included the 7 direct targets reported in PrCa (MMP2, DEDD2, NDRG2, AR, LMNB1, PARVA, SLC8A1) and 16 direct targets empirically validated in other cancers compiled in TarBase (RUNX3, TP53INP1, PPARG, CSF1, UVRAG, ZEB1, DICER1, STAT3, PDGFRA, ZBTB4, PTEN, SMAD4, ITGB1, CCNA2, PPARGC1A, FMR1 for miR-130b and TP63 and DNMT1 for miR-301b) [61] (Table 2). It is worth to note that TarBase failed to identify all the candidate target genes with experimental evidence reported or predicted in PrCa. As expected, the genes showing higher correlation coefficients also show lower *p* values (Table 2) (individual miRNA-mRNA scatter plots are available in Additional file 4: Figure S4 and Additional file 5: Figure S5). Highly significant negative correlations (*p* < 0.0001) are only produced by PrCa TSG, yielding coefficients between − 0.35 and − 0.21. Correlations between 0.05 > *p*>0.001 have lower *r* values (− 0.14 to − 0.1) and are shown by both TSG and OG. Interestingly, the three highly significant (*p* < 0.0001) positively correlated genes are OGs. Among the 4 highly significant predicted targets in PrCa, three (NDRG1, SLC8A1 and PARVA) show negative while one (LMNB1) shows a positive association with the miRNAs in TCGA-PRAD. Meanwhile, oncogenic MMP2, proposed to be repressed by the cluster in a study in PrCa, shows a weak negative correlation with the miRNAs (*r* − 0.11 *p *= 0.01 for miR-130b and *r* − 0.13 *p *= 0.03 for miR-301b). Finally, the expression of the other 2 experimentally proposed targets in PrCa (tumor suppressor AR and oncogenic DEDD2) does not correlate with the miRNA cluster. In order to establish a reference framework for the interpretation of these results, we calculated the correlation for miRNA-mRNA target pairs with strong empirical evidence in PrCa. Supporting the relevance of the associations identified for miR-130b/301b in the TCGA-PRAD, we found *r* values below − 0.21 for highly significant targets (*p *< 0.0001 yield by 9 out of the 11 evaluated) (Additional file 6: Table S1).

**Table 2:** Association between the expression of miR-130b/301b and putative direct target genes in TCGA-PRAD

Gene	PMID	Role in PrCa	miR-130b		miR-301b	
			*r*	*p* value	*r*	*p* value
*PPARGC1A*	*23868745*	*TSG? (PMC4884178)*	*− 0.3571*	*< 0.0001*	*− 0.3109*	*< 0.0001*
*TP63* ^a^	*24398967*	*TSG*	*− 0.3313*	*< 0.0001*	*− 0.2286*	*< 0.0001*
*NDRG2* ^b^	*27327120*	*TSG*	*− 0.2985*	*< 0.0001*	*− 0.2427*	*< 0.0001*
*SLC8A1* ^c^	*26489476*	*TSG*	*− 0.2884*	*< 0.0001*	*− 0.3174*	*< 0.0001*
*PARVA* ^c^	*26489476*	*TSG*	*− 0.2846*	*< 0.0001*	*− 0.3113*	*< 0.0001*
*SMAD4*	*24220575*	*TSG*	*− 0.2240*	*< 0.0001*	*− 0.2151*	*0.0002*
*ZBTB4*	*24220575*	*TSG*	*− 0.2231*	*< 0.0001*	*− 0.2829*	*< 0.0001*
*ITGB1*	*24498407*	*TSG*	*− 0.2119*	*< 0.0001*	*− 0.1995*	*0.0006*
STAT3	24040078	OG	**− **0.1423	0.0009	− 0.2049	0.0004
ZEB1	22847613	OG	**− **0.1338	0.0018	− 0.2058	0.0004
FMR1	24021279	unknown	**− **0.1250	0.0035	− 0.1380	0.0175
CSF1	22005523	OG	**− **0.1190	0.0055	− 0.1904	0.001
PTEN	24220575	TSG	**− **0.1174	0.0062	− 0.1072	0.0654
MMP2^b^	25154741	OG	**− **0.1094	0.0108	− 0.1286	0.027
PPARG	21135128	TSG	**− **0.1072	0.0125	− 0.0358	0.5397
PDGFRA	22995917	OG	**− **0.1041	0.0152	− 0.1640	0.0047
UVRAG	22228303	TSG	**− **0.0812	0.0585	− 0.0984	0.0909
RUNX3	20176475	TSG	**− **0.0220	0.6099	− 0.1161	0.046
AR^b^	28192399	TSG	**− **0.0201	0.6405	− 0.0772	0.1856
DEDD2^b^	27307749	TSG	0.0080	0.8522	0.0418	0.4733
DICER1	23392577	unknown	0.1111	0.0096	0.0297	0.6112
*TP53INP1*	*18974142*	*OG*	*0.2378*	*< 0.0001*	*0.1746*	*0.0026*
*LMNB1* ^b^	*28166834*	*OG*	*0.2404*	*< 0.0001*	*0.2071*	*0.0003*
*CCNA2*	*24681352*	*OG*	*0.2947*	*< 0.0001*	*0.2004*	*0.0005*

Correlation between miRNAs and validated mRNA targets gene expression. For each target gene the role in PrCa, the literature reference (PMID) and the correlation *r* and *p* value are shown

Qualifiers in column 1 indicate: ^a^miR-301b direct target with strong experimental evidence assigned by TarBase. DNMT1 was listed by TarBase but not included in this list since the referenced article demonstrates the absence of effect of miR-301b on DNMT1

^b^Direct target genes with experimental validation in PrCa

^c^Direct Targets predicted in PrCa. Absence of qualifier in column one indicates directed targets with strong experimental evidence assigned. TarBase does not identify targets for miR-130b/301b in PrCa. Correlations with *p* < 0.0001 are highlighted in italics

## Discussion

Lately, independent PrCa studies have analyzed the expression of miR-130b/301b cluster or the individual miRNAs derived from it. Although the majority of them found an upregulation of the miRNAs in neoplastic vs normal tissue, some studies report its downregulation, which is proposed to be due to an increased methylation of the gene in the neoplastic tissue [29]. Caution must be taken when comparing the profiling studies since the control samples are heterogeneous (normal tissue adjacent to tumor or adjacent to bladder tumor, normal tissue from normal donors, BPH), thus the disagreement in the differentially expressed miRNAs might only rely on the nature of the samples compared. Independent groups studied the effect of miR-130b/301b cluster on PrCa cell phenotype achieving contradictory results. Indeed, a tumor suppressor function has been proposed for miR-130b [29, 52] and miR-301b [29], which were shown to inhibit PrCa invasion, homing or cell cycle progression. Only the latter report [29] studied both members of the cluster in PrCa, albeit not testing their simultaneous action. Meanwhile, an oncogenic role has been proposed for miR-130b [38, 44, 62] and miR-301b [63] in PrCa, which were revealed to promote cell proliferation, viability, migration, invasion or tumor initiating properties. These conflicting findings may be explained by the use of different cell line models, different molecules to alter the level of miRNAs and diverse readouts for the functional assays employed. These discrepancies rise a warning about the lack of consensus regarding the methodologies and gold standards used in current miRNA research.

In the era of massive genomic data, the analysis of public patient gene expression data is a fundamental resource to understand the definite importance of a gene for a disease. Although miR-130b/301b has been previously measured in several PrCa cohorts, the largest and most comprehensive PrCa cohort publicly available, the TCGA-PRAD, has not been interrogated yet. We then analyzed this dataset for associations between the genomic status and expression of miR-130b/301b cluster and several aspects of the disease. We confirmed an upregulation of both miR-130b and miR-301b in tumor vs normal tissue in TCGA-PRAD specimens. Likewise, the expression of miR-130b positively correlates with clinical parameters such us T stage, residual tumor and primary therapy outcome. The failure of miR-301b to associate with clinical variables may be due to its reduced expression in prostatic tissue relative to miR-130b. Although cluster miRNAs are transcribed from the same primary transcripts, there are frequently expressed at different levels due to still poorly understood post-transcriptional regulation [64, 65]. Overall, the association between the expression of miR-130b/301b cluster and PrCa evolution in TCGA-PRAD strongly support an oncogenic action of this miRNA cluster in the disease.

The analysis of the genomic and epigenomic features of the miR-130b/301b cluster revealed its active transcription in PrCa cells. A modest increase in the DNA methylation of the locus in PrCa has been recently shown in a Portuguese PrCa cohort [29]. We were able to validate their finding in the TCGA-PRAD. Nevertheless, contradicting our findings on TCGA-PRAD (Fig. 3b, c), the authors proposed that the increased promoter methylation of the cluster is responsible for the repression of its expression. The accessibility of the miR-130b/301b CpG island, as well as its unmethylated status in PrCa (β-value < 0.2 [66]) determined by our study, argue against its silencing by DNA methylation. In addition, the small increase in CpG methylation observed in neoplastic tissue is likely insufficient to provoke the silencing of the gene (β-value remains under 0.2). In addition, larger alterations in DNA methylation from normal to tumor tissue have been reported for validated PrCa TSGs as GSTP1 [67]. Nevertheless, the most relevant finding is the positive correlation between DNA methylation and miR-130b/301b expression in TCGA-PRAD, which demonstrates that the methylation of this locus is not causing its repression in PrCa. On the contrary, since this locus is preferentially methylated at the gene body, and this region is known to stimulate transcription elongation [68], it is tempting to speculate that the molecular etiology of the upregulation of miR-130b/301b in PrCa is the increase in DNA methylation at the gene body.

The role of the cluster miR-130b/301b in PrCa carcinogenesis has been addressed by several groups. It was proposed that miR-301b expression is induced in vitro by hypoxia in PrCa cell lines (DU145, PC-3, LNCaP) causing an increase of autophagy, leading to the loss of radio-sensitivity [51]; the same report proposed tumor suppressive hydrolase NDRG2 as a direct target of miR-301b. In a functional screening of gain of function of miRNAs in 5 PrCa cell lines, Aakula et al. identified miR-130b in a group of 14 miRNAs that increase PrCa cell proliferation and change consistently its expression in clinical samples; using Taylor et al. dataset, they found that only miR-130b associates with patient survival and increases cell viability while reducing apoptosis [44]. The authors proposed that the actin-binding protein PARVA and SLC8A1 are possible direct targets of the miRNA. A later study confirmed miR-130b upregulation in an independent cohort, and demonstrated its positive influence on cell viability and its negative influence on apoptosis in LNCaP and PC-3 cell lines, reversing the effect of luteolin [59]. They also showed evidence favoring its direct repression of the proapoptotic protein DEDD2. Recently, further proof of an oncogenic role of miR-130b has been provided by Cannistraci et al. [38], who showed its impact in PrCa cell invasion in vitro and in vivo (22-Rv1, C41IM and LNCaP), and its ability to directly repress the expression of the androgen receptor AR, thus increasing the resistance to androgen therapy. Interestingly, miR-130b was shown to be present in CA-24 exosomes (in comparison to RWPE-1), and their uptake induced PrCa patient adipose-derived stem cells (pASC) neoplastic reprogramming through the upregulation of hRAS, kRAS and the downregulation of TSG PDCD4 [69]. Nevertheless, two independent groups proposed a tumor suppressor role of the miR-130b/301b cluster in PrCa. Firstly, Chen et al., reported the downregulation of miR-130b in PrCa and present evidence in favor of its ability to inhibit PrCa cell migration (in M12 and P69 cell lines) and in vitro invasion (in PC-3 cells). Indeed, they proved its capacity to repress the expression of oncogenic matrix metalloprotease MMP2 in vitro [52]. Secondly, Ramalho-Carvalho et al. found an expression profile and a function consistent with a tumor suppressor role of miR-130b and miR-301b in PrCa, showing their ability to reduce cell viability, induce DNA damage, apoptosis and cell senescence [29]. They propose LMNB1 as a candidate direct target gene of the miRNA cluster.

The availability of large patient cohorts with both small RNA and mRNA data enables to find support of the role of a miRNA through the study of its negative correlation with candidate target genes (i.e., gene repression). Due to the conflictive reports about the role of miR-130b/301b in PrCa, both OGs and TSGs have been proposed as targets of repression. However, when we analyzed the correlation between the miRNAs-target pairs proposed in the PrCa literature in the TCGA-PRAD cohort, we only found highly significant (*p* < 0.0001) negative and positive correlations with TSGs and OGs respectively, which provides additional indication of the oncogenic role of the miR-130b/301b cluster in PrCa. In particular, of the 7 genes that have been proposed as direct targets in PrCa, 4 show statistically significant correlations (*p* < 0.0001) that support an oncogenic action of the miRNAs. The magnitude of the significant correlations found for these miRNA-mRNA pairs on the TCGA-PRAD is similar to that observed for strongly evidenced pairs in PrCa. The positive correlation of LMNB1 and miR-130b/301b expression (also observed by TP53INP1 and CCNA2) may be caused by a non-canonical effect of the miRNAs on these targets. A group of miRNA-mRNA interactions activate the transcription or the translation of the target mRNA; they are mediated by direct miRNA binding to the promoter/5′UTR or the 3′UTR respectively (reviewed in [70, 71]). Since LMNB1, as well as TP53INP1 and CCNA2, lacks miR-130b/301b binding sites at their promoters it is tempting to speculate that miR-130b/301b may be stimulating the translation of these mRNA causing the stabilization of their transcripts [72]. Meanwhile, the other 3 PrCa candidate targets do not (AR, DEDD2) or weakly associate (MMP2) with the miRNA levels. Overall, the analysis of correlations for miRNA-mRNA pairs in the TCGA-PRAD favors the repression of TSGs, supporting an oncogenic action of the miR-130b/301b cluster in prostate. It is worth to mention that conclusions derived from this type of study are limited by the lack of sensitivity for miRNA translational repression not leading to a change in mRNA stability. In addition, the failure of the analysis to confirm some of the experimentally validated mRNA targets proposed in the literature might be explained by the interplay of non-miRNA mediated regulatory steps (including transcription, processing, decay factors, ncRNAs) which may override miRNAs´ regulation in patient tissue. Given the current availability of large public cohorts, our analyses highlight the relevance of the study of the clinical set to drive conclusions about the importance of miRNA/mRNA targets for a disease.

## Conclusion

The expression profile of miR-130b and miR-301b, their correlation with candidate TSG targets, as well as their association with PrCa aggressiveness in the TCGA-PRAD support an oncogenic function for these miRNAs for the disease. Most of the previous studies using independent cohorts support the same hypothesis. Although the increased methylation of the locus in cancer relative to normal tissue predicts a decrease in its expression in tumors and therefore a possible TSG function, the positive correlation between the expression of the miRNAs and the DNA methylation of the locus argues against its epigenetic repression by DNA methylation. Our study reinforces the importance of the exhaustive interrogation of the large genomic information currently available in PrCa for the evaluation of miRNA relevance in this neoplasia.

## Additional files

**Additional file 1: Figure S1.** Detailed genomic view of the miR-130b/301b gene cluster region in UCSC Genome browser (GRCh37/hg19). Several ENCODE tracks are displayed.

**Additional file 2: Figure S2.** Pattern of DNA methylation of the miR-130b/miR-301b locus in prostate datasets. Methylation levels (beta-value) of the 12 CpG dinucleotide probes located along the gene obtained using the Infinium HumanMethylation450 BeadCHiP array. The beta-value of methylation of each site and the standard deviation of the measurements are indicated. The ratio of fluorescence intensity between the unmethylated and methylated sites ranges between 0 and 1 respectively. Grey and black circles correspond to normal and tumor tissue respectively. Horizontal boxes indicate the position of the CpG island, S-shore, precursor miRNAs and POLR2A (RNA Polymerase II). A. 52 normal and 52 matched tumor samples from GSE76938 [30]. B. 5 normal and 25 unmatched tumor samples from GSE38240 [28]. C. 4 normal and 8 matched metastatic tumor samples from GSE52955 [29]. *p <0.05; **p <0.01; ***p <0.001; ****p <0.0001; *ns* non-significant.

**Additional file 3: Figure S3.** Pattern of DNA methylation of the miR-130b/miR-301b locus in prostate cell lines. Methylation levels (beta-value) of the 12 CpG dinucleotide probes located along the gene obtained using the Infinium HumanMethylation450 BeadCHiP array of PrCa cell lines GSE34340, GSE62053, GSE54758 [31, 32]. The beta-value of methylation of each site is indicated. The ratio of fluorescence intensity between the unmethylated and methylated sites ranges between 0 and 1 respectively. Horizontal boxes indicate the position of the CpG island, S-shore, precursor miRNAs and POLR2A (RNA Polymerase II).

**Additional file 4: Figure S4.** Correlations between miR-130b and target mRNAs expression in TCGA-PRAD. Scatter plots for target mRNAs highlighted in bold in Table 2, with negative (A) and positive (B) correlations. The non-parametric Spearman correlation coefficient (*r*) is indicated.

**Additional file 5: Figure S5.** Correlation between miR-301b and target mRNAs expression in TCGA-PRAD. Scatter plots for target mRNAs highlighted in bold in Table 2, with negative (A) and positive (B) correlations. The non-parametric Spearman correlation coefficient (*r*) is indicated.

**Additional file 6: Table S1.** Correlation between the expression of validated miRNA-mRNA target gene pairs in TCGA-PRAD. For each gene target the role in PrCa, reference indicated as PMID, correlation *r* and *p* value are shown. No qualifier- miR-130b directed targets with strong experimental evidence assigned by TarBase. a- miR-301b direct targets with strong experimental evidence assigned by TarBase. †-Direct Targets with experimental validation in PrCa which are not identified by TarBase. *-Direct Targets predicted in PrCa which are not identified by TarBase.

## Abbreviations

Chr: chromosome
miRNA: microRNA
miR-130b: hsa-miR-130b-3p
miR-301b: hsa-miR-301b-3p
OG: oncogene
PRAD: prostate adenocarcinoma
PrCa: prostate cancer
RPM: reads per million
TCGA: The Cancer Genome Atlas
TSG: tumor suppressor gene
