# Supplementary figures and images for: An integrated view of the role of miR-130b/301b miRNA cluster in prostate cancer
Source: Exp Hematol Oncol. 2018 May 2;7:10. doi: 10.1186/s40164-018-0102-0 (PMC5930504; doi:10.1186/s40164-018-0102-0)

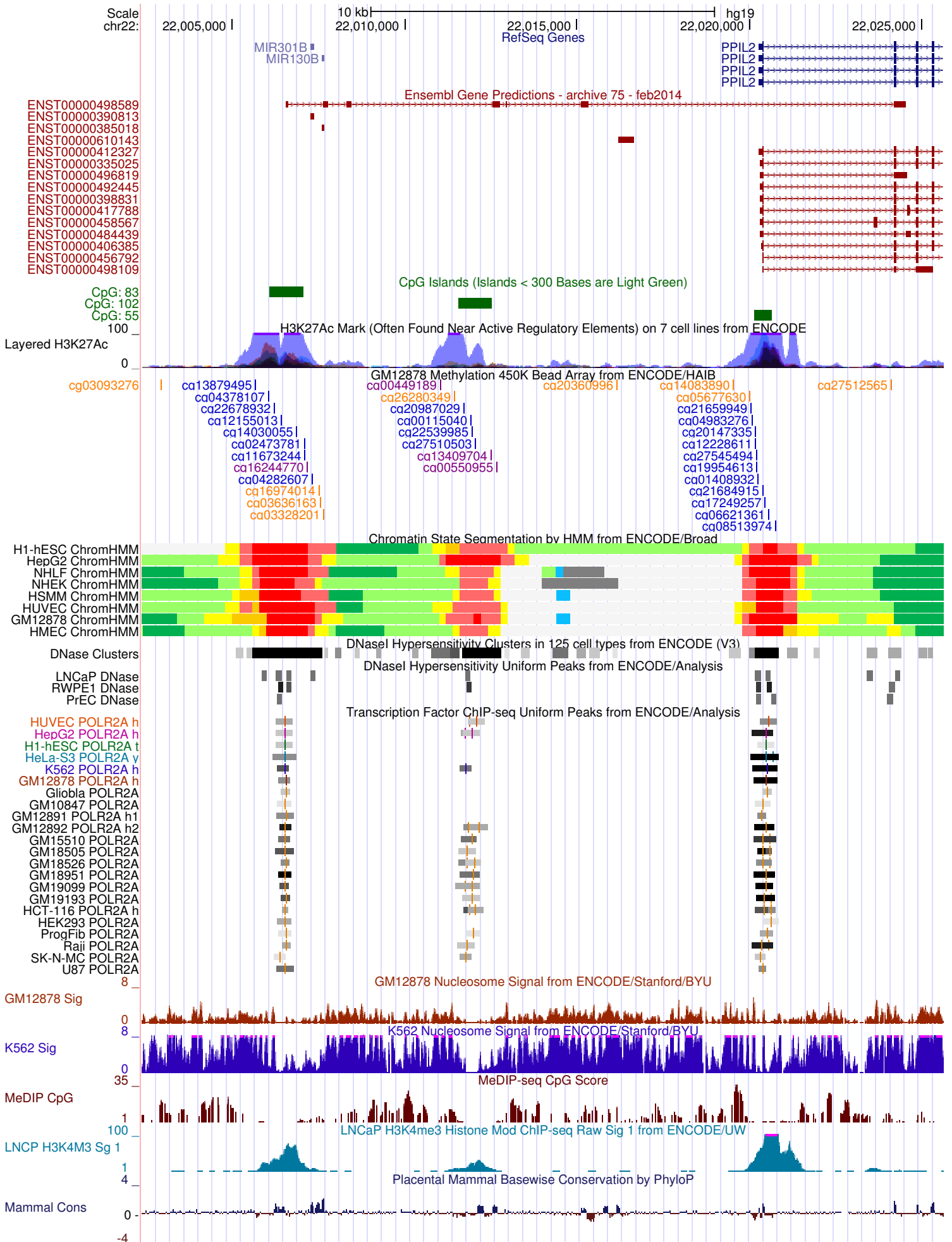

Supplement: Supplementary file 1 — Additional file 1: Figure S1. Detailed genomic view of the miR-130b/301b gene cluster region in UCSC Genome browser (GRCh37/hg19). Several ENCODE tracks are displayed. [file 40164_2018_102_MOESM1_ESM.pdf]

**A**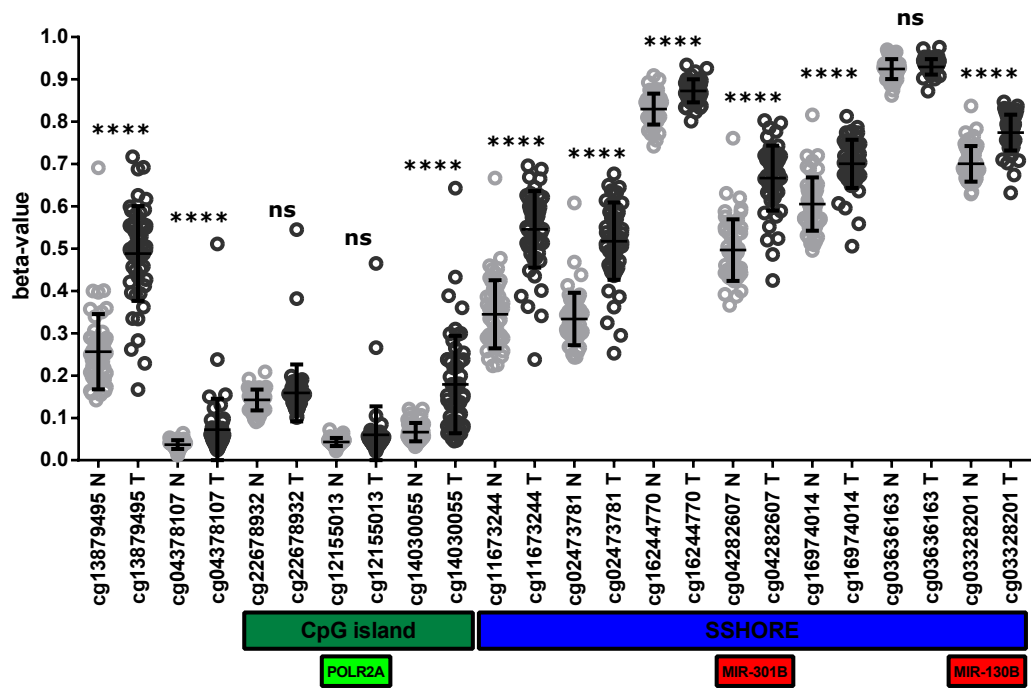**B**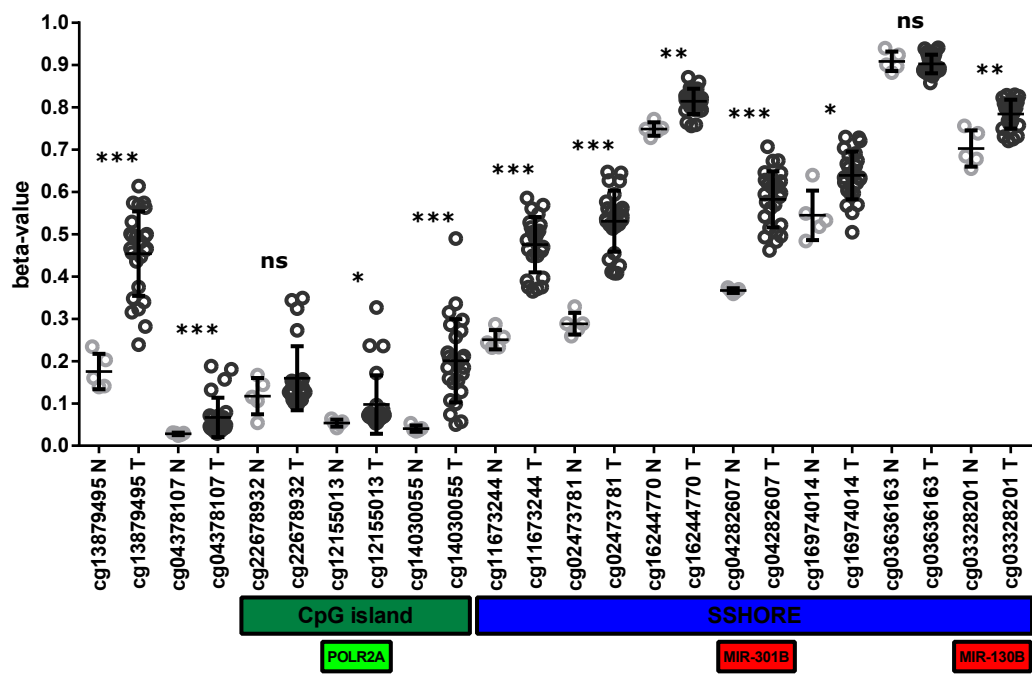**C**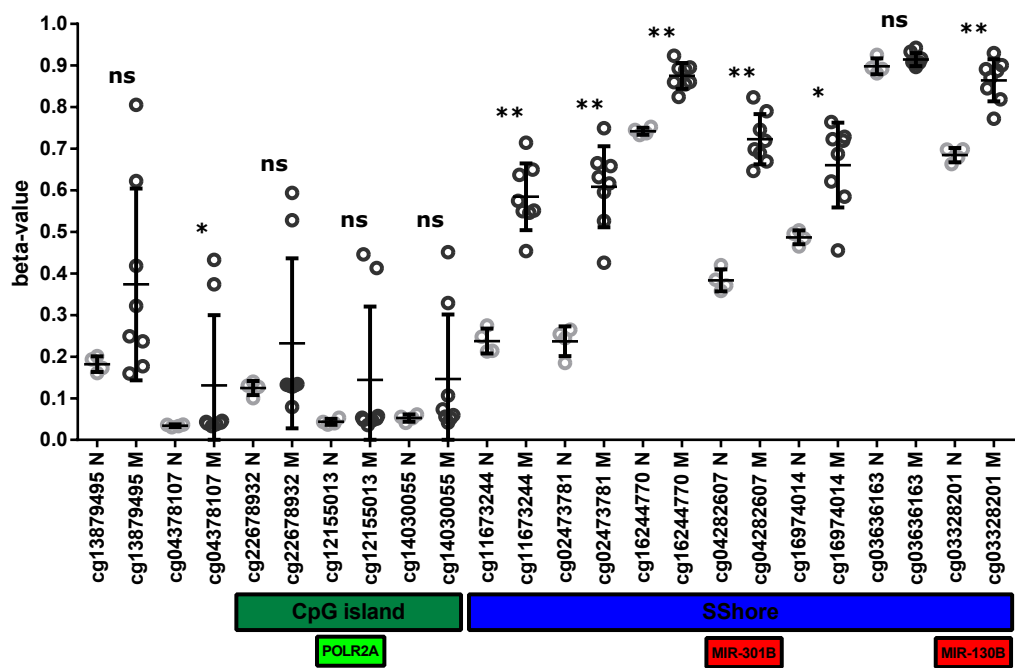

Supplement: Supplementary file 2 — Additional file 2: Figure S2. Pattern of DNA methylation of the miR-130b/miR-301b locus in prostate datasets. Methylation levels (beta-value) of the 12 CpG dinucleotide probes located along the gene obtained using the Infinium HumanMethylation450 BeadCHiP array. The beta-value of methylation of each site and the standard deviation of the measurements are indicated. The ratio of fluorescence intensity between the unmethylated and methylated sites ranges between 0 and 1 respectively. Grey and black circles correspond to normal and tumor tissue respectively. Horizontal boxes indicate the position of the CpG island, S-shore, precursor miRNAs and POLR2A (RNA Polymerase II). A. 52 normal and 52 matched tumor samples from GSE76938 [30]. B. 5 normal and 25 unmatched tumor samples from GSE38240 [28]. C. 4 normal and 8 matched metastatic tumor samples from GSE52955 [29]. *p <0.05; **p <0.01; ***p <0.001; ****p <0.0001; ns non-significant. [file 40164_2018_102_MOESM2_ESM.pdf]

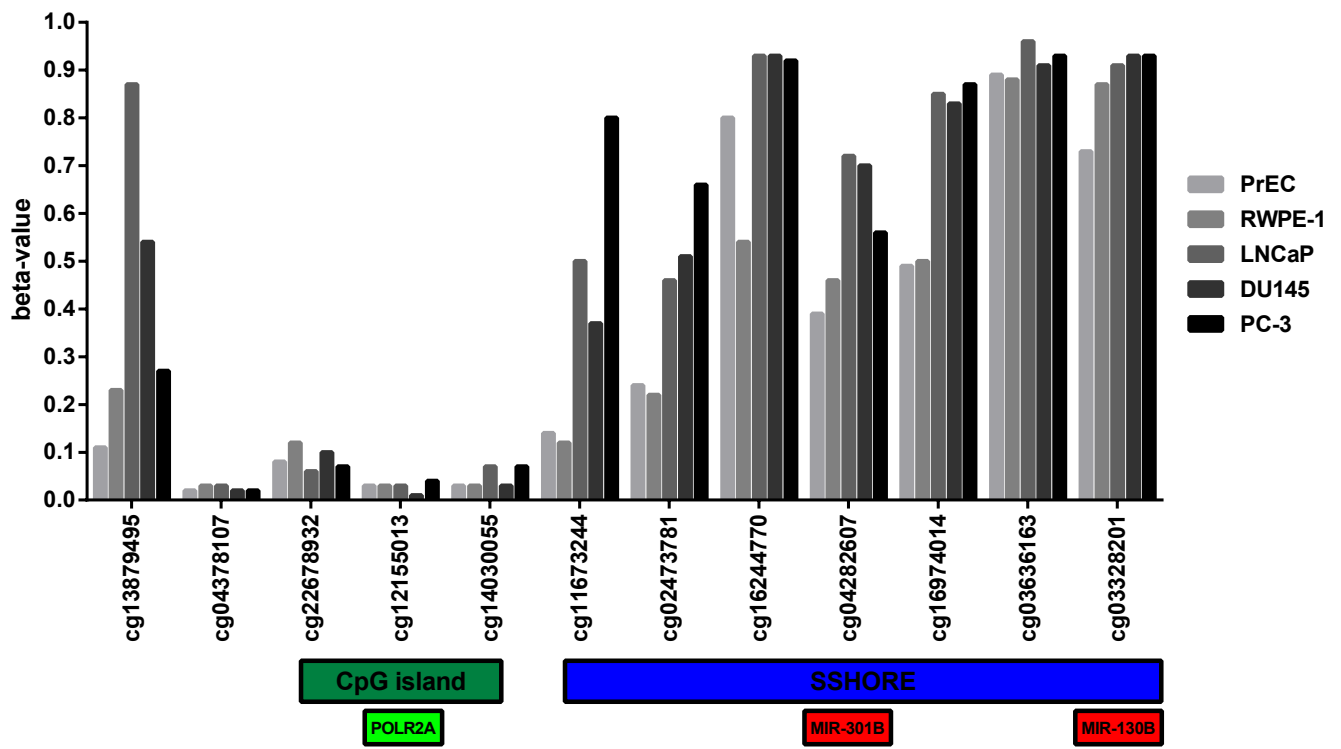

Supplement: Supplementary file 3 — Additional file 3: Figure S3. Pattern of DNA methylation of the miR-130b/miR-301b locus in prostate cell lines. Methylation levels (beta-value) of the 12 CpG dinucleotide probes located along the gene obtained using the Infinium HumanMethylation450 BeadCHiP array of PrCa cell lines GSE34340, GSE62053, GSE54758 [31, 32]. The beta-value of methylation of each site is indicated. The ratio of fluorescence intensity between the unmethylated and methylated sites ranges between 0 and 1 respectively. Horizontal boxes indicate the position of the CpG island, S-shore, precursor miRNAs and POLR2A (RNA Polymerase II). [file 40164_2018_102_MOESM3_ESM.pdf]

A

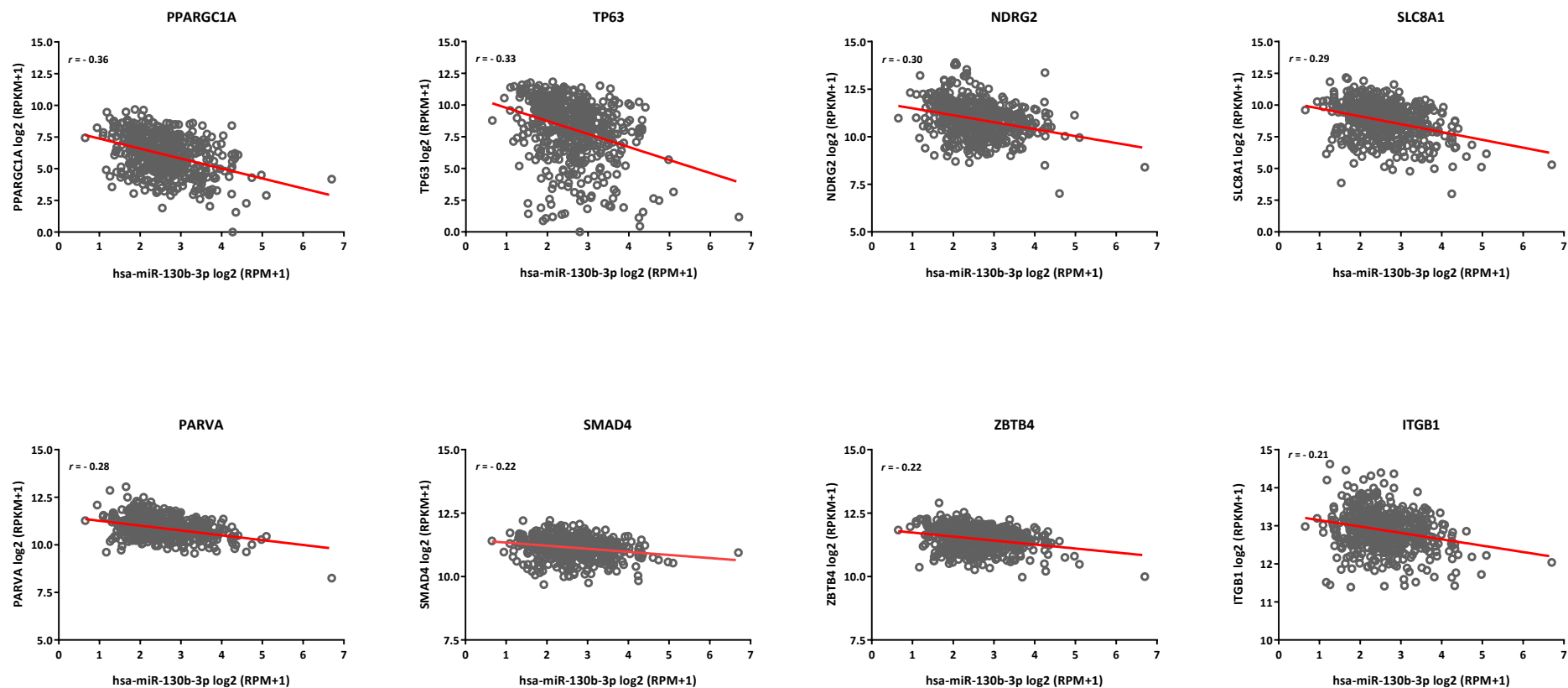

B

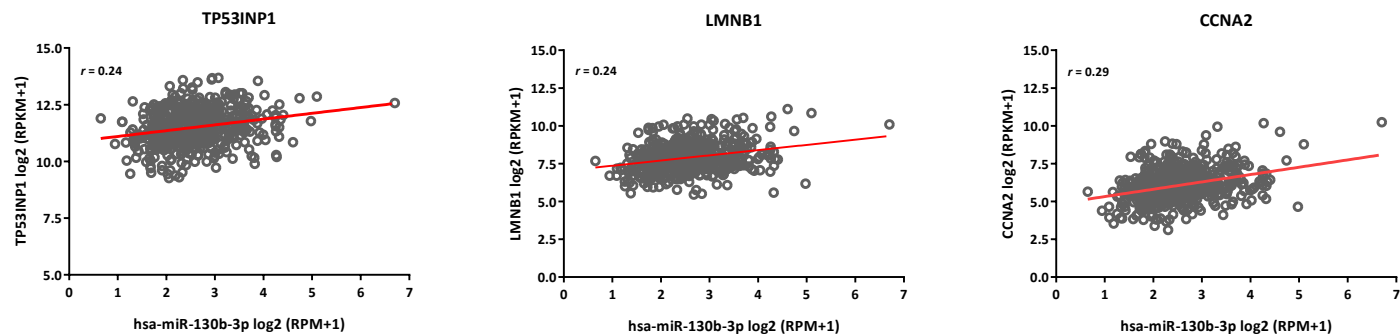

Supplement: Supplementary file 4 — Additional file 4: Figure S4. Correlations between miR-130b and target mRNAs expression in TCGA-PRAD. Scatter plots for target mRNAs highlighted in bold in Table 2, with negative (A) and positive (B) correlations. The non-parametric Spearman correlation coefficient (r) is indicated. [file 40164_2018_102_MOESM4_ESM.pdf]

A

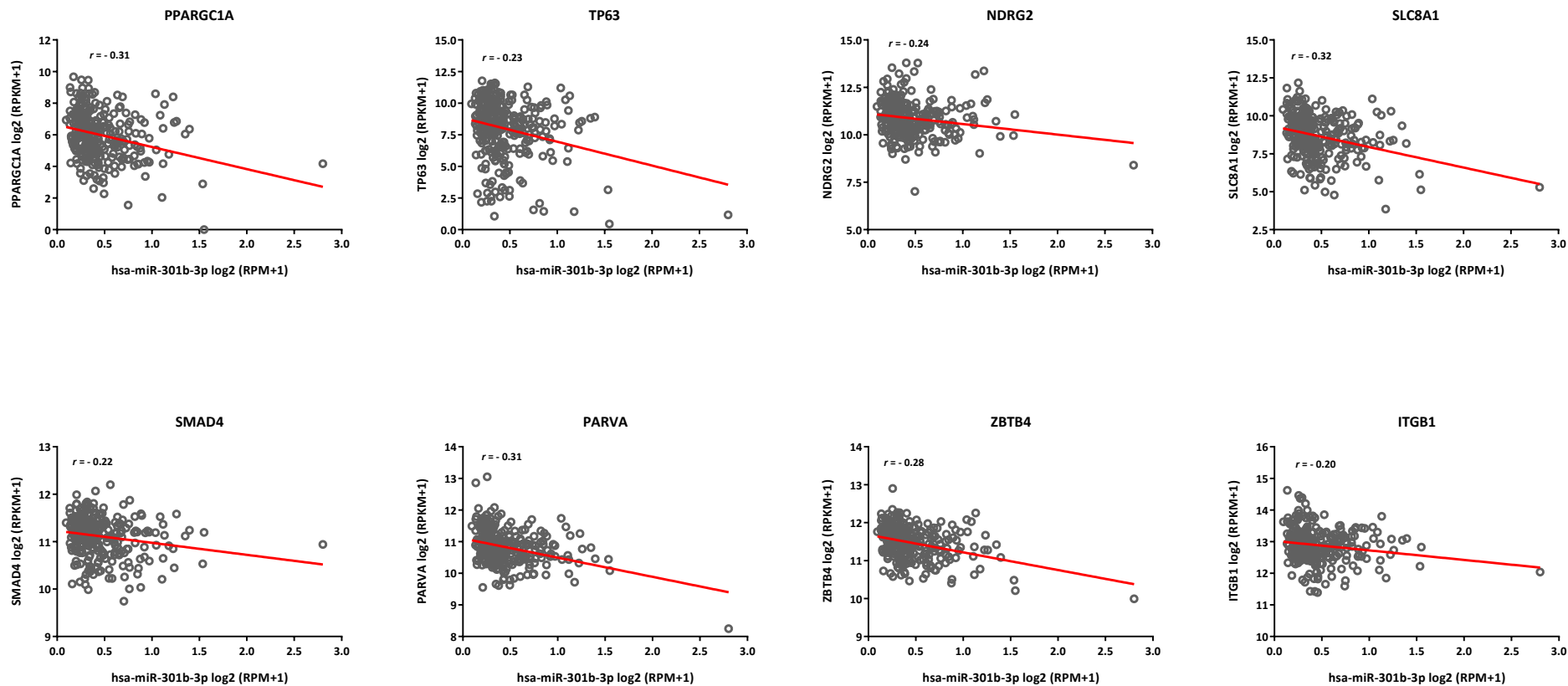

B

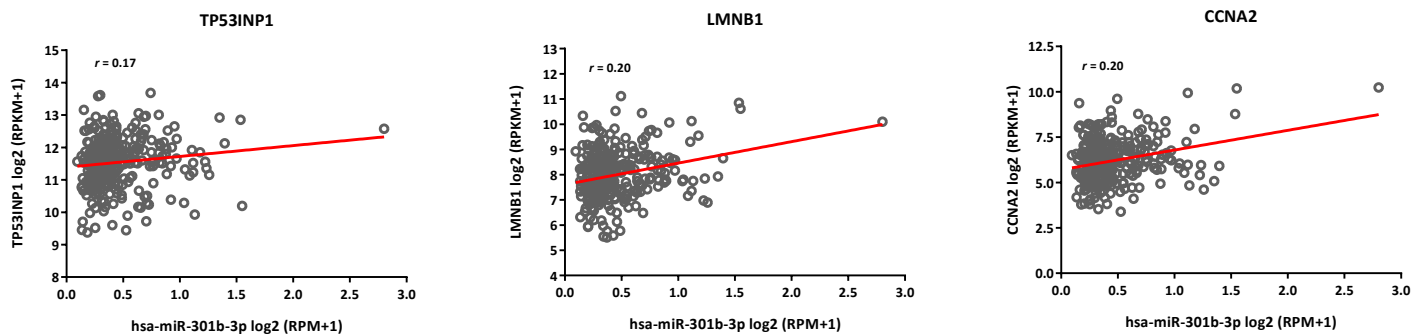

Supplement: Supplementary file 5 — Additional file 5: Figure S5. Correlation between miR-301b and target mRNAs expression in TCGA-PRAD. Scatter plots for target mRNAs highlighted in bold in Table 2, with negative (A) and positive (B) correlations. The non-parametric Spearman correlation coefficient (r) is indicated. [file 40164_2018_102_MOESM5_ESM.pdf]
